# Evolution and refinement of magnetically guided sentinel lymph node detection in breast cancer: meta-analysis

**DOI:** 10.1093/bjs/znac426

**Published:** 2022-12-23

**Authors:** Eirini Pantiora, Marios Konstantinos Tasoulis, Antonios Valachis, Staffan Eriksson, Thorsten Kühn, Andreas Karakatsanis, Isabel T Rubio

**Affiliations:** Department for Surgical Sciences, Uppsala University, Uppsala, Sweden; Section for Breast Surgery, Department of Surgery, Uppsala University Hospital, Uppsala, Sweden; Breast Surgery Unit, Royal Marsden NHS Foundation Trust, London, UK; Division of Breast Cancer Research, Institute of Cancer Research, London, UK; Department of Oncology, Örebro University Hospital, School of Medicine, Örebro University, Örebro, Sweden; Section for Breast Surgery, Department of Surgery, Västmanland Hospital, Västerås, Sweden; Centre for Clinical Research, Uppsala University, Västerås, Sweden; Department of Gynaecology and Obstetrics, Interdisciplinary Breast Centre, Hospital Esslingen, Esslingen, Germany; Department for Surgical Sciences, Uppsala University, Uppsala, Sweden; Section for Breast Surgery, Department of Surgery, Uppsala University Hospital, Uppsala, Sweden; Breast Surgical Unit, Clinica Universidad de Navarra, Cancer Centre University of Navarra, Madrid, Spain

## Abstract

**Background:**

Superparamagnetic iron oxide nanoparticles (SPIO) have been used as a tracer for sentinel lymph node (SLN) localization in breast cancer, demonstrating comparable performance to the combination of radioisotope (RI) and blue dye (BD).

**Methods:**

A systematic literature search and meta-analysis with subgroup and meta-regression analysis were undertaken to update the available evidence, assess technique evolution, and define knowledge gaps. Recommendations were made using the GRADE approach.

**Results:**

In 20 comparative studies, the detection rate was 97.5 per cent for SPIO and 96.5 per cent for RI ± BD (risk ratio 1.006, 95 per cent c.i. 0.992 to 1.019; *P* = 0.376, high-certainty evidence). Neoadjuvant therapy, injection site, injection volume or nodal metastasis burden did not affect the detection rate, but injection over 24 h before surgery increased the detection rate on meta-regression. Concordance was 99.0 per cent and reverse concordance 97.1 per cent (rate difference 0.003, 95 per cent c.i. −0.009 to 0.015; *P* = 0.656, high-certainty evidence). Use of SPIO led to retrieval of slightly more SLNs (pooled mean 1.96 *versus* 1.89) with a higher nodal detection rate (94.1 *versus* 83.5 per cent; RR 1.098, 1.058 to 1.140; *P* < 0.001; low-certainty evidence). In meta-regression, injection over 24 h before surgery increased the SPIO nodal yield over that of RI ± BD. The skin-staining rate was 30.8 per cent (very low-certainty evidence), and possibly prevented with use of smaller doses and peritumoral injection.

**Conclusion:**

The performance of SPIO is comparable to that of RI ± BD. Preoperative injection increases the detection rate and nodal yield, without affecting concordance. Whether skin staining and MRI artefacts are reduced by lower dose and peritumoral injection needs to be investigated.

## Teaser

The magnetic technique for sentinel lymph node (SLN) biopsy has gained interest and popularity in recent years. In this comprehensive systematic review and meta-analysis, the performance of the magnetic technique was comparable to that of the isotope and blue dye combination for SLN detection (97.5 *versus* 96.5 per cent; risk ratio 1.006, 95 per cent c.i. 0.992 to 1.019; *P* = 0.376). The magnetic technique provides flexibility and facilitates logistics. Further research should focus on the existing knowledge gaps, namely expansion of novel techniques such as delayed SLN biopsy and reduction of the risk of skin staining and artefacts on postoperative MRI.

## Introduction

Assessment of sentinel lymph node (SLN) status remains a significant component of breast cancer management, being routine practice in the majority of patients with a clinically negative axilla^1^. Radioisotopes (RIs) and blue dye (BD) have been the preferred tracers for SLN localization during the past two decades. This procedure, however, poses challenges not only associated with the regulations for manipulation and disposal of the radioactive materials, but also in terms of administration logistics. Conventional tracers are subject to limitations related to patient management, especially owing to the restricted time frame from injection to surgery^2^. New methods have consequently been developed to fill this gap.

Superparamagnetic iron oxide nanoparticles (SPIO) have been tested as SLN localization tracer in multiple studies and meta-analyses. Many trials^3–10^ have shown high concordance with conventional localization techniques and non-inferiority to RI ± BD regarding the detection rate. Several studies^7,10,11^ have reported skin staining, mainly after breast-conserving surgery. In addition, concerns have been raised regarding potential artefacts in postoperative MRI^12–15^. The technique has evolved in recent years, showing promising results with smaller doses of SPIO, injected not only in the subareolar region^16,17^ but also close to the tumour^18^. The efficacy of injection in different time frames has also been tested, ranging from intraoperative administration to up to several weeks before surgery^19–21^. At the same time, the introduction of paramagnetic markers for the localization of impalpable lesions^22,23^ offers the option of an integrated platform for breast and axillary procedures^24^. In this setting, the only consideration is that the use of metallic instruments interferes with the magnetic signal, and so plastic or titanium instruments need to be used instead.

The aim of this systematic review and meta-analysis was to examine the available data on SPIO in breast cancer surgery, the performance of SPIO as a tracer in SLN biopsy (SLNB), and to investigate factors associated with technique refinement. Finally, the role of the magnetic technique in addressing tailored patient needs and knowledge gaps was evaluated.

## Methods

### Endpoints

The primary endpoint for this meta-analysis was the detection rate for SPIO per patient, defined as the proportion of patients with at least one SLN detected successfully by the magnetic technique divided by the total number of patients. As a second primary endpoint, factors that influence the detection rate were investigated. Secondary endpoints were: detection rate per SLN, defined as the proportion of SLNs detected successfully by the magnetic technique divided by the total number of SLNs retrieved; SLN yield, expressed as the average (pooled mean) number of SLNs retrieved; prevalence of SPIO-induced skin staining, defined as documented skin staining after SPIO injection and associated factors; SPIO-induced artefacts in postoperative MRI; and cost-effectiveness. Finally, in comparative studies, the concordance between SPIO and RI was analysed. For the latter, concordance was defined as the proportion of the number of patients in whom SPIO and RI were both successful, divided by the number of patients in whom RI was successful.


$$\mathrm{Concordance}\;=\;\frac{SPIO\;+\;\;\;RI}{RI}$$

Reverse concordance was defined as the proportion of the number of patients in whom SPIO and RI were both successful, divided by the number of patients in whom SPIO was successful.


$$\mathrm{Reverse}\;\mathrm{concordance}\;=\;\frac{SPIO+RI}{SPIO}$$

For tracers performing in an equivalent manner, the assumption is that they should be successful in the same patients, that is N_(SPIO + RI)_ = N_SPIO_ = N_RI_, meaning that the rate difference (RD = concordance – reverse concordance) should be 0. However, if one of the two tracers performs better than another single tracer, that is, if N_RI_ ≠ N_SPIO_, then concordance rates may be high or low, although this may not be clinically relevant. Therefore, RD was selected as effect size and was retrieved from comparative studies with a paired design. Pooled proportions and risk ratios (RRs) in comparative studies, with 95 per cent confidence intervals, were calculated to express the other outcomes. In studies in which BD was used as an adjunct for both SPIO and RI, successful detection was considered with the addition of BD for both tracers.

The findings of the meta-analysis were summarized in the form of clinical questions according to the Grading of Recommendations, Assessment, Development and Evaluation (GRADE) tool^25^ by two authors. Lack of evidence in clinically relevant questions was defined as a knowledge gap after discussion among the authors.

### Literature search

A PubMed and MEDLINE search was performed using the search terms ‘magnetic technique’, ‘superparamagnetic iron oxide nanoparticles’, ‘sentinel lymph node’, ‘breast cancer’ according to the PRISMA statement^26^. A parallel search of other literature sources, including abstracts from congress volumes and citation searches, was undertaken. Authors of source studies were contacted for additional data, if deemed necessary. Single-arm, prospective, and retrospective cohort studies, and comparative, randomized and non-randomized trials were included if they provided data on the primary endpoint of the meta-analysis. For comparative trials, an isotope tracer was required as control. Any studies comparing SPIO with exclusive use of BD were excluded. Preclinical data, studies with fewer than 10 participants, and studies reporting on systems that were not available commercially at the time of publication were excluded. The literature search ended in February 2022.

### Data extraction and analyses

Included studies were screened independently by two authors and the data were stored in a preformed worksheet (Microsoft^®^ Excel; Microsoft, Redmond, WA, USA). The DerSimonian Laird random-effects model was selected *a priori*^27^. Reported effect sizes were calculated from the results of the entire source study and leave-one-out meta-analyses were performed for sensitivity. Separate analyses for detection rates and in the presence of metastasis were undertaken for the available comparative studies. Heterogeneity was evaluated by means of the *I*^2^ statistic^28^. Subgroup and meta-regression analyses were performed for type of SPIO, type of probe, dose of SPIO, timing of SLNB (upfront or after neoadjuvant therapy), site of injection (subareolar or periareolar *versus* peritumoral) and timing of injection (perioperative, suggesting intraoperative and less than 24 h before surgery; preoperative, more than 24 h before surgery). For this, studies reporting on distinct subgroups were split into respective subgroups. Publication bias was examined by inspection of funnel plots and Egger’s test for small studies effect^29^. Meta-analyses were undertaken in Stata^®^ release 17 (StataCorp, College Station, TX, USA). For pooled rates of proportions, such as detection rates and skin staining, single-arm studies of SPIO and the SPIO arm of comparative trials were analysed using the metaprop command^30^. For these studies, meta-regression was performed with the metareg command^31^.

### Bias assessment

The Risk Of Bias In Non-randomized Studies of Interventions (ROBINS-I)^32^ and Methodological Index for Non-Randomized Studies (MINORS)^33^ tools were used to assess bias in the included comparative studies. Single-arm studies were assessed using the MINORS tool for single-arm studies. The observational studies addressing MRI outcomes were assessed by means of the Newcastle–Ottawa Scale (NOS) for cohort studies^34^, and the quality assessment tool for diagnostic accuracy studies (QUADAS-2) for studies of diagnostic accuracy^35^. These assessments were carried out by two authors and consensus was reached after discussion. For the studies reporting on detection rates, the MINORS version was selected for the manuscript, for uniformity of presentation and the conduct of meta-regression analyses that would allow insight on whether reported outcomes might be affected by study quality.

## Results

The systematic literature review identified 32 studies that were appropriate for inclusion in qualitative and quantitative synthesis (*Fig. S1*). Twenty studies^3–11,16–20,36–42^ were comparative (SPIO *versus* RI ± BD), of which 19 undertook concomitant administration of SPIO and RI ± BD in the same patients (paired design), whereas 7 were non-comparative^24,43–48^. Of these, two trials^10,18^ overlapped as the study by Hersi *et al.*^18^ was a patient-level meta-analysis including the outcomes of Karakatsanis *et al.*^10^. The overlapping patient group was removed from the study by Hersi *et al.*^18^, to avoid duplication. Three studies^36,40,48^ presented dedicated data on SLNB after neoadjuvant treatment, but only one^40^ reported clearly on the original nodal status. Furthermore, one study^41^ was used only to discuss discolouration data, and four^12–15^ were dedicated to reporting MRI artefacts. There was only one randomized trial^17^, which compared different doses of SPIO; no other randomized trials comparing SPIO with RI ± BD could be retrieved. Finally, one trial (SentiNot)^19^ examined the role of SPIO in the context of delayed SLNB, in patients initially operated for ductal carcinoma *in situ* (DCIS). In this study, SPIO was injected peritumorally in the breast during the breast procedure and the patient was taken to delayed SLNB in another session, only if underlying invasive cancer was found in the specimen. The RI was injected before delayed SLNB in the previous excision site and the subareolar region or, in the event of mastectomy, intradermally near the scar or the areola^19^. All included studies are summarized in *Tables 1* and *2*, with the respective MINORS and NOS scores for study quality. A detailed assessment of study quality and the risk of bias assessed using MINORS and ROBINS-I for studies reporting on detection rates, and NOS and QUADAS-2 for studies reporting on MRI artefacts, is available in *Table S1*.

**Table 1:** Characteristics of the comparative studies included in the systematic review and meta-analysis

Reference	Data accrual	SLNB procedures	SPIO	Injection volume (ml)	Injection site	Timing of injection	MINORS score
Douek *et al.*^3^	Prospective, non-randomized	160	Sienna+^®^	2 ml SPIO diluted in 3 ml NaCl or local anaesthetic	Subareolar	Peroperative	23
Thill *et al.*^4^	Prospective, non-randomized	150	Sienna+^®^	2 ml SPIO diluted in 3 ml NaCl or local anaesthetic	Subareolar	Peroperative	22
Rubio *et al.*^36^‡	Prospective, non-randomized	30	Sienna+^®^	2 ml SPIO diluted in 3 ml NaCl or local anaesthetic	Subareolar	Peroperative	20
Rubio *et al.*^5^	Prospective, non-randomized	120	Sienna+^®^	2 ml SPIO diluted in 3 ml NaCl or local anaesthetic	Subareolar	Peroperative	23
Piñeiro-Madrona *et al.*^6^	Prospective, non-randomized	181	Sienna+^®^	2 ml SPIO diluted in 3 ml NaCl or local anaesthetic	Subareolar	Peroperative	24
Ghilli *et al.*^7^	Prospective, non-randomized	197	Sienna+^®^	2 ml SPIO diluted in 3 ml NaCl or local anaesthetic	Subareolar	Peroperative	24
Coufal *et al.*^8^	Prospective, non-randomized	20	Sienna+^®^	2 ml SPIO diluted in 3 ml NaCl or local anaesthetic	Subareolar	Peroperative	20
Ahmed *et al.*^37^	Prospective, non-randomized	32	Sienna+^®^	0.5 ml SPIO	Peritumoral	Peroperative	21
Houpeau *et al.*^9^	Prospective, non-randomized	108	Sienna+^®^	2 ml SPIO diluted in 3 ml NaCl or local anaesthetic	Subareolar	Peroperative	24
Karakatsanis *et al.*^10^	Prospective, non-randomized	206	Sienna+^®^	2 ml SPIO diluted in 3 ml NaCl or local anaesthetic	Subareolar	Peroperative	24
Karakatsanis *et al.*^11^*	Prospective, non-randomized	339	Sienna+^®^	2 ml SPIO diluted in 3 ml NaCl or local anaesthetic	Subareolar/peritumoral	Peroperative/preoperative	24
Karakatsanis *et al.*^20^	Prospective, non-randomized	12	Sienna+^®^	2 ml SPIO diluted in 3 ml NaCl or local anaesthetic	Subareolar	Preoperative	22
Karakatsanis *et al.*^19^§	Prospective, non-randomized	40	Sienna+^®^	2 ml SPIO	Subareolar/peritumoral	Preoperative	24
Alvarado *et al.*^16^	Prospective, non-randomized	146	Magtrace^®^	2 ml SPIO	Subareolar	Peroperative	24
Taruno *et al.*^38^†	Prospective, non-randomized	210	Ferucarbutran	1 ml SPIO	Subareolar	Peroperative	24
Makita *et al.*^42^†	Prospective, non-randomized	69	Ferucarbutran	0.5 ml SPIO	Subareolar/peritumoral	Peroperative	22
Hamzah *et al.*^39^	Prospective, non-randomized	20	Magtrace^®^	2 ml SPIO	Subareolar	Peroperative	20
Rubio *et al.*^17^	Prospective, randomized	135	Magtrace^®^	SPIO in different doses	Subareolar	Peroperative	24
Hersi *et al.*^18^	Prospective, non-randomized	328	Magtrace^®^	SPIO in different doses	Subareolar/peritumoral	Peroperative/preoperative	24
Giménez-Climent *et al.*^40^‡	Prospective, non-randomized	89	Sienna+^®^	2 ml SPIO diluted in 3 ml NaCl or local anaesthetic	Subareolar	Peroperative	22
Wärnberg *et al.*^41^¶	Prospective, observational	340	Sienna+^®^	2 ml SPIO diluted in 3 ml NaCl or local anaesthetic	Subareolar/peritumoral	Peroperative/preoperative	20

Head-to-head comparison between superparamagnetic iron oxide nanoparticles (SPIO) and radioisotope. All other studies had a within-patient comparison design—all patients received both tracers ± blue dye and paired comparisons were made. †Used Tokyo probe; the Sentimag^®^ system (Endomag, Cambridge, UK) was used in all other studies. ‡Sentinel lymph node biopsy (SLNB) after neoadjuvant treatment. §Delayed SLNB after primary surgery for ductal carcinoma *in situ* (SentiNot study). ¶Reported only skin-staining outcomes. MINORS, Methodological Index for Non-Randomized Studies. Sienna+^®^ (Endomag, Campbridge, UK); Magtrace^®^ (Endomag, Cambridge, UK).

**Table 2:** Characteristics of the non-comparative studies included in the systematic review and meta-analysis

Reference	Data accrual	Procedures	SPIO	Injection volume (ml)	Injection site	Timing of injection	Study quality score
**Detection rate and skin staining**							
ȃHersi *et al.*^24^‡	Prospective, observational	32 SLNB	Magtrace^®^	2 ml SPIO	Peritumoral	Peroperative	14
ȃLorek *et al.*^43^§	Retrospective	303 SLNB	Sienna+^®^	2 ml SPIO diluted in 3 ml NaCl or local anaesthetic	Subareolar	Peroperative	11
ȃMan *et al.*^44^	Retrospective	333 SLNB	Magtrace^®^	2 ml SPIO	Subareolar	Peroperative	13
ȃVural and Yilmaz^45^	Prospective, observational	104 SLNB	Sienna+^®^	2 ml SPIO diluted in 3 ml NaCl or local anaesthetic	Subareolar	Peroperative	14
ȃBazire *et al.*^46^¶	Retrospective	288 SLNB	Sienna+^®^	2 ml SPIO diluted in 3 ml NaCl or local anaesthetic	Subareolar	Peroperative	9
ȃPohlodek *et al.*^47^‡	Retrospective	38 SLNB	Sienna+^®^	2 ml SPIO diluted in 3 ml NaCl or local anaesthetic	Subareolar	Peroperative	13
ȃKurylcio *et al.*^48^#	Retrospective	76 SLNB	Sienna+^®^	2 ml SPIO diluted in 3 ml NaCl or local anaesthetic	Subareolar	Peroperative	10
**SPIO artefacts on postoperative MRI**							
ȃKrischer *et al.*^12^	Retrospective	23 MRI	Sienna+^®^	2 ml SPIO diluted in 3 ml NaCl or local anaesthetic	Subareolar	Peroperative	3†
ȃAribal *et al.*^13^	Retrospective	36 MRI	Sienna+^®^	2 ml SPIO diluted in 3 ml NaCl or local anaesthetic	Subareolar	Peroperative	3†
ȃChapman *et al.*^14^	Retrospective	21	Sienna+^®^	2 ml SPIO diluted in 3 ml NaCl or local anaesthetic	Subareolar	Peroperative	3†
ȃChristenhusz *et al.*^15^	Retrospective	76	Sienna+^®^	2 ml SPIO in 3 ml NaCl subareolar or 0.1 ml intratumoral	Subareolar/intratumoral	Peroperative	5†

Methodological Index for Non-Randomized Studies score, except †Newcastle–Ottawa Scale score. ‡Examined the combination of superparamagnetic iron oxide nanoparticles (SPIO) with a paramagnetic seed for tumour localization. §Primary endpoint was complications of SPIO sentinel lymph node dissection (SLND) procedures. ¶Primary endpoint was the safety of postoperative radiotherapy after SPIO SLND procedures. #SLND after neoadjuvant treatment. SLNB, sentinel lymph node biopsy.

### Detection rate

The pooled SLN detection rate for SPIO across all studies (27 in total, 20 comparative and 7 non-comparative) was 98.7 (95 per cent c.i. 98.1 to 99.2) per cent, with low heterogeneity (*I*^2^ = 25.0 per cent, *P* = 0.119). For this outcome, meta-regression analysis showed that a lower MINORS score was significantly associated with higher reported detection rates (exp(b) = 0.9992, 95 per cent c.i. 0.9982 to 0.9998; *P* = 0.013; *I*^2^ = 16.9 per cent). Across 20 comparative studies, the pooled detection rate was 97.5 (96.8 to 98.1) per cent for SPIO and 96.5 (95.7 to 97.2) per cent for RI ± BD, but the difference was not significant (RR 1.006, 95 per cent c.i. 0.992 to 1.019; *P* = 0.376; *I^2^* = 28.7 per cent) (*Fig. S2*). The results were independent of pN status. For pN+ disease, across 16 comparative studies the pooled detection rate was 99.4 (97.8 to 100) per cent for SPIO and 97.0 (92.8 to 99.7) per cent for RI ± BD, indicating comparable performance (RR 1.006, 0.982 to 1.031; *P* = 0.637; *I*^2^ = 0 per cent). Leave-one-out meta-analysis did not affect the results.

Subgroup analyses showed that probe type, SPIO type, SPIO dose, neoadjuvant therapy, and type of study design did not influence outcomes, whereas peritumoral injection was associated with a trend for better detection for SPIO over RI ± BD. SPIO demonstrated improved detection over RI ± BD after preoperative injection and in the setting of SentiNot, which examined the feasibility of delayed SLNB. These effects were retained on meta-regression analysis. There was no heterogeneity (*I^2^* = 0 per cent). The results are summarized in *Table 3*.

**Table 3:** Subgroup and meta-regression analysis examining factors for successful superparamagnetic iron oxide nanoparticle-guided sentinel lymph node detection

	Subgroup analysis		Meta-regression analysis	
	Risk ratio	*P*	Coefficient b	*P*
**Probe**				
ȃSentimag^®^	1.007 (0.994, 1.019)	0.289		
ȃTokyo probe	1.003 (0.924, 1.088)	0.941		
**SPIO**				
ȃFerucarbutran	1.003 (0.924, 1.088)	0.941		
ȃMagtrace^®^	1.010 (0.992, 1.029)	0.277		
ȃSienna + ^®^	1.004 (0.988, 1.021)	0.598		
**Injection site**			–0.0083 (–0.0663, 0.0498)*	0.781
ȃSubareolar	1.000 (0.991, 1.010)	0.957		
ȃPeritumoral	1.118 (0.982, 1.272)	0.091		
**Timing of injection**			0.0544 (0.0042, 0.1045)†	0.034
ȃPeroperative	0.999 (0.990, 1.008)	0.819		
ȃPreoperative	1.116 (1.020, 1.222)	0.017		
**Setting**				
ȃAfter neoadjuvant therapy	1.021 (0.975, 1.069)	0.375		
ȃUpfront	1.005 (0.992, 1.018)	0.442		
**Comparison**				
ȃPaired	1.007 (0.995, 1.020)	0.251		
ȃUnpaired	0.956 (0.893, 1.024)	0.197		
**Subgroup**			–0.3627 (–0.5967, –0.1287)‡	0.002
ȃStandard SLNB	1.002 (0.993, 1.011)	0.661		
ȃDelayed SLNB	1.528 (1.216, 1.922)	< 0.001		
**Injection volume (ml)**				
ȃ0.5	1.032 (0.981, 1.086)	0.227		
ȃ1.0	1.016 (0.968, 1.067)	0.522		
ȃ1.5	0.988 (0.959, 1.017)	0.407		
ȃ2.0	1.042 (0.974, 1.115)	0.231		
ȃ5.0	1.000 (0.988, 1.013)	0.959		

Values in parentheses are 95% confidence intervals. Coefficient for *subareolar injection, †preoperative injection or ‡standard sentinel lymph node dissection. SPIO, superparamagnetic iron oxide nanoparticles; SLNB, sentinel lymph node biopsy.

### Nodal retrieval and nodal detection rate

Data from 24 studies were available for this analysis. In crude analysis, the pooled mean number of SLNs retrieved per procedure with the magnetic technique was 2.3. The pooled nodal detection rate was 96.0 (95 per cent c.i. 93.5 to 98.1) per cent, but the results were highly heterogeneous (*I*^2^ = 95.3 per cent). No subgroup analyses were attempted.

Across 19 comparative studies, the nodal detection rate was significantly higher for SPIO than for RI ± BD (94.1 (91.8 to 96.1) *versus* 83.5 (78.7 to 87.9) per cent; RR 1.098, 95 per cent c.i. 1.058 to 1.140; *P* < 0.001), but with marked heterogeneity (*I*^2^ = 85.2 per cent) (*Fig. S3*). Leave-one-out meta-analysis did not change the outcome. However, crude pooled analysis showed that this difference was not clinically relevant when examining the pooled mean number of SLNs identified and excised for SPIO and RI ± BD (1.93 *versus* 1.85 respectively). In meta-regression analysis, use of the Sentimag^®^ probe, preoperative SPIO injection, SLND after neoadjuvant therapy, and delayed SLNB were associated with a higher nodal detection rate for SPIO over RI ± BD (*Table 4*). Type of SPIO, SPIO dose, SPIO injection site, and type of study (paired *versus* non-paired comparative) were not significant. There was high heterogeneity (*I*^2^ = 70.0 per cent) and the Egger test demonstrated a small studies effect (β1 = 1.83, *P* < 0.001), which mandates that these findings are interpreted with caution.

**Table 4:** Subgroup and meta-regression analysis of nodal detection rate

	Subgroup analysis		Meta-regression analysis	
	Risk ratio	*P*	Coefficient b	*P*
**Probe**			0.3566 (0.1641, 0.5490)*	<0.001
ȃSentimag^®^	1.072 (1.038, 1.108)	<0.001		
ȃTokyo probe	1.456 (1.318, 1.608)	<0.001		
**SPIO**			−0.0019 (−0.1593, 0.1555)	0.981
ȃFerucarbutran	1.456 (1.318, 1.608)	<0.001		
ȃMagtrace^®^	1.066 (1.010, 1.126)	0.021		
ȃSienna+^®^	1.078 (1.032, 1.126)	0.001		
**Injection site**			0.0395 (−0.1173, 0.1963)	0.622
ȃPeritumoral	1.303 (1.008, 1.683)	0.043		
ȃSubareolar	1.082 (1.043, 1.122)	<0.001		
**Timing of injection**			0.1473 (0.0371, 0.2574)†	0.009
ȃPreoperative	1.284 (1.095, 1.507)	0.002		
ȃPeroperative	1.070 (1.033, 1.109)	<0.001		
**Setting**			−0.1179 (−0.2231, −0.0127)‡	0.028
ȃUpfront	1.078 (1.039, 1.118)	<0.001		
ȃAfter neoadjuvant therapy	1.308 (0.894, 1.912)	0.167		
**Comparison**			−0.1258 (−0.2685, 0.0169)	0.084
ȃPaired	1.097 (1.057, 1.139)	<0.001		
ȃUnpaired	0.930 (0.841, 1.029)	0.158		
**Subgroup**			−0.8075 (–1.2011, –0.4139)§	<0.001
ȃStandard SLNB	1.080 (1.044, 1.118)	<0.001		
ȃDelayed SLNB	2.571 (1.788, 3.699)	<0.001		
**Injection volume (ml)**			0.0092 (–0.0365, 0.05489)	0.693
ȃ0.5	1.188 (0.796, 1.772)	0.400		
ȃ1.0	1.128 (1.053, 1.207)	0.001		
ȃ1.5	1.001 (0.926, 1.083)	0.970		
ȃ2.0	1.175 (1.025, 1.346)	0.021		
ȃ5.0	1.069 (1.031, 1.110)	<0.001		

Values in parentheses are 95% confidence intervals. Coefficient for *Tokyo probe, †preoperative injection, ‡upfront surgery or §standard sentinel lymph node biopsy. SPIO, superparamagnetic iron oxide nanoparticles; SLNB, sentinel lymph node biopsy.

### Concordance

Only 19 studies with a paired design were appropriate for examination of concordance. The pooled concordance rate ($\frac{SPIO+\;RI}{RI}$) was 99.0 (95 per cent c.i. 98.2 to 99.6) per cent (*I*^2^ = 34.2 per cent, *P* = 0.073) and the reverse concordance rate ($\frac{SPIO+\;RI}{SPIO}$) was 97.1 (95.2 to 98.6) per cent (*I*^2^ = 75.0 per cent, *P* < 0.001). The pooled difference was −0.003 (95 per cent c.i. −0.009 to 0.015; *P* = 0.656), with moderate heterogeneity (*I*^2^ = 59.5 per cent) (*Fig. S4*). Leave-one-out meta-analysis did not affect this outcome. In subgroup and meta-regression analysis, concordance was not affected by any factor. Reverse concordance, as expected, was decreased by the factors that increased SPIO detection over RI ± BD, subsequently affecting the RD. Indeed, subgroup and meta-regression analysis for the difference verified that preoperative SPIO injection and delayed SLN biopsy (SLNB) (SentiNot) detection affected this outcome (*Table 5* and *Fig. S4*). The very high collinearity between SPIO detection and reverse concordance, however, limits the size of explained variance by the meta-regression model. Indeed, the adjusted *R*^2^ value was 0 per cent, suggesting that the difference between concordance and reverse concordance probably stems from the fact that the detection rate was higher with use of SPIO than with RI ± BD for preoperative SPIO detection and the SentiNot technique.

**Table 5:** Subgroup and meta-regression analysis for rate difference (concordance – reverse concordance)

	Subgroup analysis		Meta-regression analysis	
	Rate difference	*P*	Coefficient b	*P*
**Probe**				
ȃSentimag^®^	−0.002 (−0.009, 0.004)	0.502		
ȃTokyo probe	−0.004 (−0.084, 0.076)	0.922		
**SPIO**				
ȃFerucarbutran	−0.004 (−0.084, 0.076)	0.922		
ȃMagtrace^®^	−0.010 (−0.022, 0.001)	0.078		
ȃSienna+^®^	−0.004 (−0.021, 0.013)	0.642		
**Injection site**				
ȃPeritumoral	−0.120 (−0.020, 0.063)	0.099		
ȃSubareolar	0.001 (−0.006, 0.007)	0.846		
**Timing of injection**			−0.0558 (−0.0904, −0.0212)*	0.002
ȃPreoperative	−0.122 (−0.219, −0.025)	0.014		
ȃPeroperative	0.001 (−0.005, 0.008)	0.655		
**Setting**				
ȃUpfront	−0.002 (−0.009, 0.004)	0.454		
ȃAfter neoadjuvant therapy	0.021 (−0.020, 0.063)	0.312		
**Subgroup**			0.2957 (0.1440, 0.4473)†	<0.001
ȃStandard SLNB	−0.0001 (−0.007, 0.006)	0.890		
ȃDelayed SLNB	−0.350 (−0.498, −0.202)	<0.001		
**Injection volume (ml)**				
ȃ0.5	−0.035 (−0.080, 0.011)	0.132		
ȃ1.0	−0.016 (−0.067, 0.034)	0.524		
ȃ1.5	0.011 (−0.022, 0.044)	0.516		
ȃ2.0	−0.011 (−0.024, 0.003)	0.121		
ȃ5.0	−0.001 (−0.007, 0.010)	0.518		

Values in parentheses are 95% confidence intervals. Coefficient for *preoperative injection or †standard sentinel lymph node dissection. SPIO, superparamagnetic iron oxide nanoparticles; SLNB, sentinel lymph node biopsy.

### Skin staining and MRI artefacts

Data for skin staining were available in 12 studies^5,7,9,10,13–15,33,36,38,40,44^ with a maximum follow-up of 3 years. The prevalence of skin staining was 30.8 (95 per cent c.i. 21.2 to 41.2) per cent, but ranged from 0 to 84.4 per cent, with very high heterogeneity (*I*^2^ = 96 per cent) (*Fig. S5*). Skin staining was reported almost exclusively (over 95 per cent) after breast-conserving surgery. In subgroup analysis, the lowest discolouration rates came with a lower SPIO dose, peritumoral injection, and preoperative injection without the need to massage. No significant associations could be demonstrated on meta-regression analysis for each factor separately, suggesting that reducing skin staining is probably best achieved by a combination of these factors (*Table 6*). Two studies^17,41^ included patient-reported outcomes, which showed that the majority of patients did not consider staining to be a problem.

**Table 6:** Subgroup and meta-regression analysis for superparamagnetic iron oxide nanoparticle-induced skin staining

	Skin staining (%)	Coefficient b	*P*
**Injection volume (ml)**		−0.0061 (−0.0816, 0.0693)*	0.863
ȃ0.5	0 (0, 19.9)		
ȃ1.0	25.2 (9.4, 41.0)		
ȃ1.5	43.3 (37.0, 49.5)		
ȃ2.0	32.1 (26.9, 37.2)		
ȃ5.0	31.3 (20.5, 42.1)		
**Injection site**		0.2390 (−0.2178, 0.6958)†	0.279
ȃSubareolar	36.6 (25.8, 47.3)		
ȃPeritumoral	15.4 (0, 36.2)		
**Massage**		0.0344 (–0.4202, 0.4891)‡	0.873
ȃYes	34.7 (22.7, 46.6)		
ȃNo	24.4 (6.3, 42.6)		

Values in parentheses are 95% confidence intervals. Coefficient for *lower superparamagnetic iron oxide nanoparticle volume, †subareolar injection or ‡perioperative injection with massage.

Four retrospective^12–15^ reports with a total of 97 patients were available on MRI artefacts after SPIO-guided SLND. The results were pooled from the source studies to analyse the role of SPIO dose, injection site, and type of surgery, stratified per study. Apart from six patients who received an intratumoral injection of 0.1 ml, all others had received 2 ml SPIO in a total volume of 5 ml in the subareolar area. Artefacts were present in 61 (95 per cent c.i. 50 to 70) per cent up to 46 months after SPIO administration. In univariable analyses, artefacts were more common after breast-conserving surgery than mastectomy (70 *versus* 21 per cent; difference 49 (95 per cent c.i. 28 to 70) per cent; *P* < 0.001). For the six patients with a 0.1-ml intratumoral SPIO injection, the incidence of MRI artefact was 0 per cent, compared with 65 per cent after a subareolar injection of 2 ml SPIO and 3 ml sodium chloride (difference 65 (55.0 to 75) per cent; *P* = 0.003). In an analysis of artefacts after breast-conserving surgery (78 patients), the effect was similar (0 *versus* 76 per cent; difference 76 (67 to 86) per cent; *P* < 0.001). Aggregated artefact rates ranged from 46 to 100 per cent among studies, owing to small numbers, high level of selection bias, and significant heterogeneity (*I*^2^ = 90 per cent). In terms of qualitative and quantitative artefact characteristics, the studies used different, non-standardized classifications, which precluded any further analyses.

### Health economic outcomes

Three studies reported on health economic outcomes. In an exploratory analysis from the Swedish MONOS trial^11^, switching from RI to SPIO would result in an average procedure-related cost reduction of €27 (€252 to €225; reduction 10.7 (95 per cent c.i. 7.2 to 15.2) per cent), whereas with preoperative, in-office SPIO administration, the average savings were €352.7 per procedure, owing to avoidance of nuclear medicine charges and theatre delays. A pilot study from Germany^49^ also showed that SPIO-guided SLNB shortened the preoperative care pathway without affecting operating time or reimbursement. The authors concluded that the technique yielded the potential to reduce costs and improve patient experience. Finally, the SentiNot interim analysis^19^ showed that, by SPIO allowing upfront SLNB to be avoided in patients with high-risk DCIS, a mean reduction of €448 (95 per cent c.i. €151 to 746) per patient, corresponding to a reduction of 8.5 per cent (€4813 *versus* 5261; *P* = 0.003), was achieved for the entire study. This reduction was even more significant for women with DCIS (and not invasive tumours) who would have undergone SLNB (mean cost saving €1296 (€3990 *versus* 5286), 24.5 per cent; *P* < 0·001). No other relevant data could be retrieved during the systematic review.

### Evidence summary, knowledge gaps, and research priorities

Summarizing the evidence according to GRADE (*Table S2*), in the setting of upfront SLNB for breast cancer, SPIO performed comparably and was concordant in terms of detection rate with RI ± BD, independently of nodal status (high-certainty evidence), retrieving slightly more SLNs (low-certainty evidence). The latter was an outcome with marked heterogeneity and may depend on other factors, such as differences in study protocols (for example registration of *ex vivo* signal with registration of more nodes as magnetic or removal of palpable lymph nodes) that are difficult to account for. Regardless, the average numbers of SLNs retrieved were similar and there should be no concern about the removal of an excessively larger number of SLNs. Interestingly, SPIO yielded a higher detection rate when administered more than 24 h before surgery, a property that should be capitalized on, as it may have the potential to provide logistical advantages, and possibly contain costs. Another point of interest from this meta-analysis is that studies with a higher risk of bias, such as retrospective analyses, and those without a control group, smaller numbers or without standardized reporting of outcomes (corresponding to a lower MINORS score), reported higher detection rates, suggesting that only well designed prospective trials are expected to improve the level of evidence for the magnetic technique.

In the present meta-analysis, skin staining after SPIO injection occurred in approximately 30 per cent of patients. The existing evidence was heterogeneous in outcomes, but also in type and duration of follow-up. Reported skin staining rates were much lower after injection of smaller volumes deep in the parenchyma and close to the tumour. The strength of recommendations is currently low owing to data heterogeneity, but, given that smaller volumes or peritumoral injection did not have adverse effects on SLN detection, this is something that should be considered. Further studies need to take these parameters into account, and provide structured follow-up and reporting of skin staining.

Regarding the presence of MRI artefacts, only retrospective reports^12–14^ were identified. It would appear that residual SPIO in the parenchyma is expected to produce artefacts in the ipsilateral breast and predominantly at the injection site. Reassuringly, the contralateral breast or other surrounding structures are not affected. The results of the meta-analysis suggest that a small injection volume in the part of the breast that will be removed may address this concern. The evidence is, however, very limited. The quality of the identified studies precludes definitive conclusions or clear recommendations. Therefore, prospective observational studies should examine the outcome of MRI artefacts in relation to different doses and injection sites, and interpret the findings in a standardized and clinically relevant manner. Currently, there are two ongoing prospective studies^17,50^ dedicated to investigating MRI artefacts after SPIO injection, one after subareolar and the other after peritumoral SPIO administration in doses of 2.0, 1.5, and 1.0 ml.

Although dedicated studies examining SPIO-guided SLND after neoadjuvant therapy were restricted, subgroup and meta-regression analyses demonstrated that SPIO performed comparably to RI in this setting. The lack of structured reports on node status before neoadjuvant therapy is a serious limitation, as no detailed conclusions can be drawn. More, well structured studies in this setting should add to the existing body of evidence. No data exist regarding the use of SPIO for SLND in pregnant patients with breast cancer, as pregnancy was an exclusion criterion in all the prospective trials identified.

## Discussion

RI ± BD has long served as the standard tracer for SLNB in patients with breast cancer. Its known restrictions, including challenging logistics, restricted access and, in the case of the dye, anaphylactic reactions, have motivated research for new techniques. The magnetic technique with SPIO is one such method. Two previous meta-analyses^10,51^ have already shown non-inferiority and reached similar conclusions, despite using different methodology. Therein, all included studies had a paired design, that is patients acted as their own controls, and all had received a perioperative subareolar injection of 5 ml (2 ml SPIO, diluted with 3 ml sodium chloride 0.9 per cent) followed by a 5-min massage. Since then, more studies have been added to the literature, evaluating SPIO as the sole tracer for SLNB, or examining the effect of different doses, injection sites in the breast, and time frames of administration. In the present meta-analysis, data synthesis verified that SPIO performs comparably to RI ± BD, regardless of dose or injection site. Both detection rates and concordance were comparable, suggesting that SPIO is a valid alternative to RI ± BD. The difference noted in nodal detection rate suggests that SPIO retrieves more SLNs, but crude analysis showed that the numerical difference is not relevant, and that SPIO-guided SLND does not result in excessive node retrieval.

A novel finding of this meta-analysis is that the preoperative injection of SPIO is not only feasible, but also increases SLN detection. Although injection more than 24 h before surgery was shown to increase detection over peroperative or intraoperative administration, the optimal or maximum interval between SPIO administration and surgery still needs to be defined. It seems that extending the time before surgery allows increased SPIO concentration in the SLN, facilitating identification, a finding in line with experimental data^52^. Several studies^11,18^ have reported on a time frame that extends up to 27 days in upfront SLNB. This has already been capitalized on in the SentiNot study, which explored the feasibility of delayed SLNB in women with a preoperative diagnosis of DCIS, in whom successful SLNB was performed up to 47 days after SPIO injection^19^. This is a property unique to SPIO and further investigation in other clinical scenarios, such as the neoadjuvant therapy setting, could provide with interesting implementations, such as SPIO administration already before the induction of neoadjuvant therapy, both in terms of clinical outcomes but also in cost containment. Recently, the feasibility of minimally invasive magnetic axillary mapping was demonstrated in the phase II MagUS study^53^, in which a group of patients were mapped with SPIO injection before neoadjuvant therapy. At surgery when SLNB or targeted axillary dissection was performed, the magnetic SLNs were still visualized on MRI, without tracer migration, and had good concordance with the isotope.

Skin staining and MRI artefacts have been the main concern regarding the SPIO technique, mostly after breast conservation. The present results suggest that staining is less with a smaller dose and a peritumoral injection can address this, as the bulk of SPIO is removed during surgery. Because there is an absolute correlation between SPIO staining and magnetic signal^10^, a similar association could be expected for MRI artefacts. The available evidence, however, stems from studies with a high risk of bias, reporting outcomes after injection of 5 ml, which is no longer used. In a study from the Netherlands^15^, it was shown that no artefacts were present in patients who had received a peritumoral, lower-volume SPIO injection. This is in line with the hypothesis that residual SPIO is related to the presence of artefacts. Therefore, removing this area should address such concerns. However, this is only a hypothesis that needs to be confirmed; currently, this topic is viewed as the most important knowledge gap to be addressed. Results from the PostMAG MRI study^50^ and the SUNRISE trial^17^ are expected to provide more insight, as these studies are examining the same question after 2.0-, 1.5-, and 1.0-ml injections, but the injection was peritumoral in PostMAG MRI and subareolar in SUNRISE. At the same time, the results suggest that further research on SPIO is required to achieve high detection rates and, at the same time, minimize the risk of skin staining and MRI artefacts.

Apart from binary meta-analyses, the magnetic technique has shown comparable performance to RI ± BD or indocyanine green in network meta-analyses^10,51,54^. However, the present work provides an updated and comprehensive review of current knowledge and provides information on the outcomes associated with use of different SPIO products, probes, doses, injection timings, and injection sites, thus contributing to the refinement of the technique. The available evidence has been evaluated according to the standardized GRADE approach, which defines the level of evidence and strength of recommendations. Interestingly, the GRADE outcomes have highlighted that, although there are no clinically relevant differences in detection rates and node retrieval between comparative and non-comparative studies, the level and strength of evidence will increase only if further research is performed in well designed prospective trials, instead of small, non-controlled studies. The latter should merely serve as pilot projects that will assess the feasibility of larger trials or report on off-label uses.

## Supplementary Material

znac426_Supplementary_Data

## Data Availability

The review protocol, template data collection forms, data extracted from included studies, and data used for all analyses can be accessed upon reasonable request.
