# Supplementary material for: Evolution and refinement of magnetically guided sentinel lymph node detection in breast cancer: meta-analysis
Source: Br J Surg. 2022 Dec 23;110(4):410–9. doi: 10.1093/bjs/znac426 (PMC10364535; doi:10.1093/bjs/znac426)
Supplement: znac426_Supplementary_Data [file znac426_supplementary_data.docx]

**Evolution and refinement of magnetic-guided sentinel lymph node detection in breast cancer: meta-analysis**

Eirini Pantiora^1,2^, Marios Konstantinos Tasoulis^3,4^, Antonios Valachis^5^, Staffan Eriksson^6,7^, Thorsten Kühn^8^, Andreas Karakatsanis^1,2 *^, Isabel T. Rubio^9 *^.

1. Department for Surgical Sciences, Uppsala University, Uppsala, Sweden.
2. Section for Breast Surgery, Department of Surgery, Uppsala University Hospital, Uppsala, Sweden.
3. Breast Surgery Unit, The Royal Marsden NHS Foundation Trust, Fulham Road, London, UK.
4. Division of Breast Cancer Research, The Institute of Cancer Research, London, UK
5. Department of Oncology, Örebro University Hospital, School of Medicine, Örebro University, Örebro, Sweden.
6. Section for Breast Surgery, Department for Surgery, Västmanland Hospital, Västerås, Sweden.
7. Centre for Clinical Research, Uppsala University, Västerås, Sweden.
8. Department of Gynaecology and Obstetrics, Interdisciplinary Breast Center, Hospital Esslingen, Esslingen, Germany.
9. Breast Surgical Unit, Clinica Universidad de Navarra, Cancer Center University of Navarra, Madrid, Spain.

*: These authors have contributed equally to the present work.

Corresponding author: Andreas Karakatsanis, Department for Surgical Sciences, Uppsala University and Section for Endocrine and Breast Surgery, Department of Surgery, Uppsala University Hospital, SE 75185, Uppsala, Sweden.

E-mail: [andreas.karakatsanis@surgsci.uu.se](mailto:andreas.karakatsanis@surgsci.uu.se),

Tel: 0046765864826. Twitter handle: @KarakatsanisA

**Supplementary Materials - Index**

| **Supplementary Figures and Tables** |  |
| --- | --- |
| Table S1: Bias assessment of the included studies in the systematic review | *pag. 3* |
| Table S2: Table of GRADE recommendations | *pag. 10* |
| Figure S1: PRISMA 2020 flow diagram for new systematic reviews which included searches of databases, registers and other sources | *pag. 11* |
| Figure S2: Forest plot for SPIO detection rate | *pag. 12* |
| Figure S3: Nodal detection Forest plot | *pag. 13* |
| Figure S4: Concordance and Reverse Concordance Forest plot | *pag. 14* |
| Figure S5: Skin Staining Rate Forest plot | *pag. 15* |
| PRISMA Checklist | *pag. 16* |
|  |  |
|  |  |

**Supplementary Tables**

**Table S1: Risk of bias assessment for the included studies**

1. **Studies on detection rate**

**1.a: Comparative studies.**

1.a.(i): Detailed MINORS criteria

| **Study ID** | **Aim** | **Inclusion** | **Prospective** | **Endpoints** | **Endpoint assessment** | **Follow-up** | **Loss <5%** | **Sample size** | **Control group** | **Contemporary**  **group** | **Group Equivalence** | **Statistical Analyses** | **Total** |
| --- | --- | --- | --- | --- | --- | --- | --- | --- | --- | --- | --- | --- | --- |
| **Douek et al, 2014 (3)** | 2 | 1 | 2 | 2 | 2 | 2 | 2 | 2 | 2 | 2 | 2 | 2 | **23** |
| **Thill et al, 2014 (4)** | 2 | 1 | 2 | 2 | 2 | 2 | 2 | 1 | 2 | 2 | 2 | 2 | **22** |
| **Rubio et al, 2014 (35)** | 2 | 2 | 2 | 2 | 2 | 1 | 2 | 0 | 2 | 2 | 2 | 1 | **20** |
| **Rubio et al, 2015 (5)** | 2 | 2 | 2 | 2 | 2 | 2 | 2 | 1 | 2 | 2 | 2 | 2 | **23** |
| **Pineiro et al, 2015 (6)** | 2 | 2 | 2 | 2 | 2 | 2 | 2 | 2 | 2 | 2 | 2 | 2 | **24** |
| **Ghilli et al, 2015 (7)** | 2 | 2 | 2 | 2 | 2 | 2 | 2 | 2 | 2 | 2 | 2 | 2 | **24** |
| **Coufal et al, 2015 (8)** | 2 | 2 | 2 | 2 | 2 | 1 | 2 | 0 | 2 | 2 | 2 | 1 | **20** |
| **Ahmed et al, 2015 (36)** | 2 | 1 | 2 | 2 | 2 | 2 | 2 | 0 | 2 | 2 | 2 | 2 | **21** |
| **Houpeau et al, 2016 (9)** | 2 | 2 | 2 | 2 | 2 | 2 | 2 | 2 | 2 | 2 | 2 | 2 | **24** |
| **Karakatsanis et al, 2016 (10)** | 2 | 2 | 2 | 2 | 2 | 2 | 2 | 2 | 2 | 2 | 2 | 2 | **24** |
| **Karakatsanis et al, 2017 (11)** | 2 | 2 | 2 | 2 | 2 | 2 | 2 | 2 | 2 | 2 | 2 | 2 | **24** |
| **Karakatsanis et al, 2018 (20)** | 2 | 2 | 2 | 2 | 2 | 2 | 2 | 0 | 2 | 2 | 2 | 2 | **22** |
| **Karakatsanis et al, 2019 (19)** | 2 | 2 | 2 | 2 | 2 | 2 | 2 | 2 | 2 | 2 | 2 | 2 | **24** |
| **Alvarado et al, 2019 (16)** | 2 | 2 | 2 | 2 | 2 | 2 | 2 | 2 | 2 | 2 | 2 | 2 | **24** |
| **Taruno et al, 2019 (37)** | 2 | 2 | 2 | 2 | 2 | 2 | 2 | 2 | 2 | 2 | 2 | 2 | **24** |
| **Makita et al, 2020 (48)** | 2 | 2 | 2 | 2 | 2 | 2 | 2 | 0 | 2 | 2 | 2 | 2 | **22** |
| **Hamzah et al, 2020 (38)** | 2 | 2 | 2 | 2 | 2 | 2 | 2 | 0 | 2 | 2 | 2 | 0 | **20** |
| **Rubio et al, 2020 (17)** | 2 | 2 | 2 | 2 | 2 | 2 | 2 | 2 | 2 | 2 | 2 | 2 | **24** |
| **Hersi et al, 2021 (18)** | 2 | 2 | 2 | 2 | 2 | 2 | 2 | 2 | 2 | 2 | 2 | 2 | **24** |
| **Giménez-Climent et al, 2021 (39)** | 2 | 2 | 2 | 2 | 2 | 2 | 2 | 0 | 2 | 2 | 2 | 2 | **22** |

1.a.(ii): ROBINS-I tool

| **Study ID** | **Confounding bias** | **Selection bias** | **Classification of interventions bias** | **Deviation from interventions bias** | **Missing data bias** | **Measurement of outcomes bias** | **Report bias** | **Overall** |
| --- | --- | --- | --- | --- | --- | --- | --- | --- |
| **Douek et al, 2014 (3)** | Low | Low | Low | Low | Moderate | Low | Low | **Low** |
| **Thill et al, 2014 (4)** | Low | Low | Low | Low | Moderate | Low | Low | **Low** |
| **Rubio et al, 2014 (35)** | Moderate | Moderate | Low | Low | Moderate | Moderate | Low | **Moderate** |
| **Rubio et al, 2015 (5)** | Low | Low | Low | Low | Low | Low | Low | **Low** |
| **Pineiro et al, 2015 (6)** | Low | Low | Low | Low | Moderate | Low | Low | **Low** |
| **Ghilli et al, 2015 (7)** | Low | Low | Low | Low | Low | Low | Low | **Low** |
| **Coufal et al, 2015 (8)** | Moderate | Moderate | Serious | Moderate | Critical | Serious | Moderate | **Moderate** |
| **Ahmed et al, 2015 (36)** | Moderate | Low | Low | Low | Low | Low | Low | **Low** |
| **Houpeau et al, 2016 (9)** | Low | Low | Low | Low | Low | Low | Low | **Low** |
| **Karakatsanis et al, 2016 (10)** | Low | Low | Low | Low | Low | Moderate | Low | **Low** |
| **Karakatsanis et al, 2017 (11)** | Low | Low | Low | Low | Low | Low | Moderate | **Low** |
| **Karakatsanis et al, 2018 (20)** | Moderate | Moderate | Moderate | Moderate | Moderate | Serious | Moderate | **Moderate** |
| **Karakatsanis et al, 2019 (19)** | Low | Moderate | Low | Low | Moderate | Low | Low | **Low** |
| **Alvarado et al, 2019 (16)** | Low | Low | Low | Low | Low | Low | Low | **Low** |
| **Taruno et al, 2019 (37)** | Low | Low | Low | Low | Low | Moderate | Low | **Low** |
| **Makita et al, 2020 (48)** | Low | Low | Moderate | Moderate | Moderate | Moderate | Low | **Moderate** |
| **Hamzah et al, 2020 (38)** | Moderate | Low | Moderate | Moderate | Moderate | Serious | Low | **Moderate** |
| **Rubio et al, 2020 (17)** | Low | Moderate | Low | Low | Low | Low | Low | **Low** |
| **Hersi et al, 2021 (18)** | Low | Low | Low | Low | Low | Low | Low | **Low** |
| **Giménez-Climent et al, 2021 (39)** | Moderate | Low | Low | Low | Low | Low | Low | **Low** |


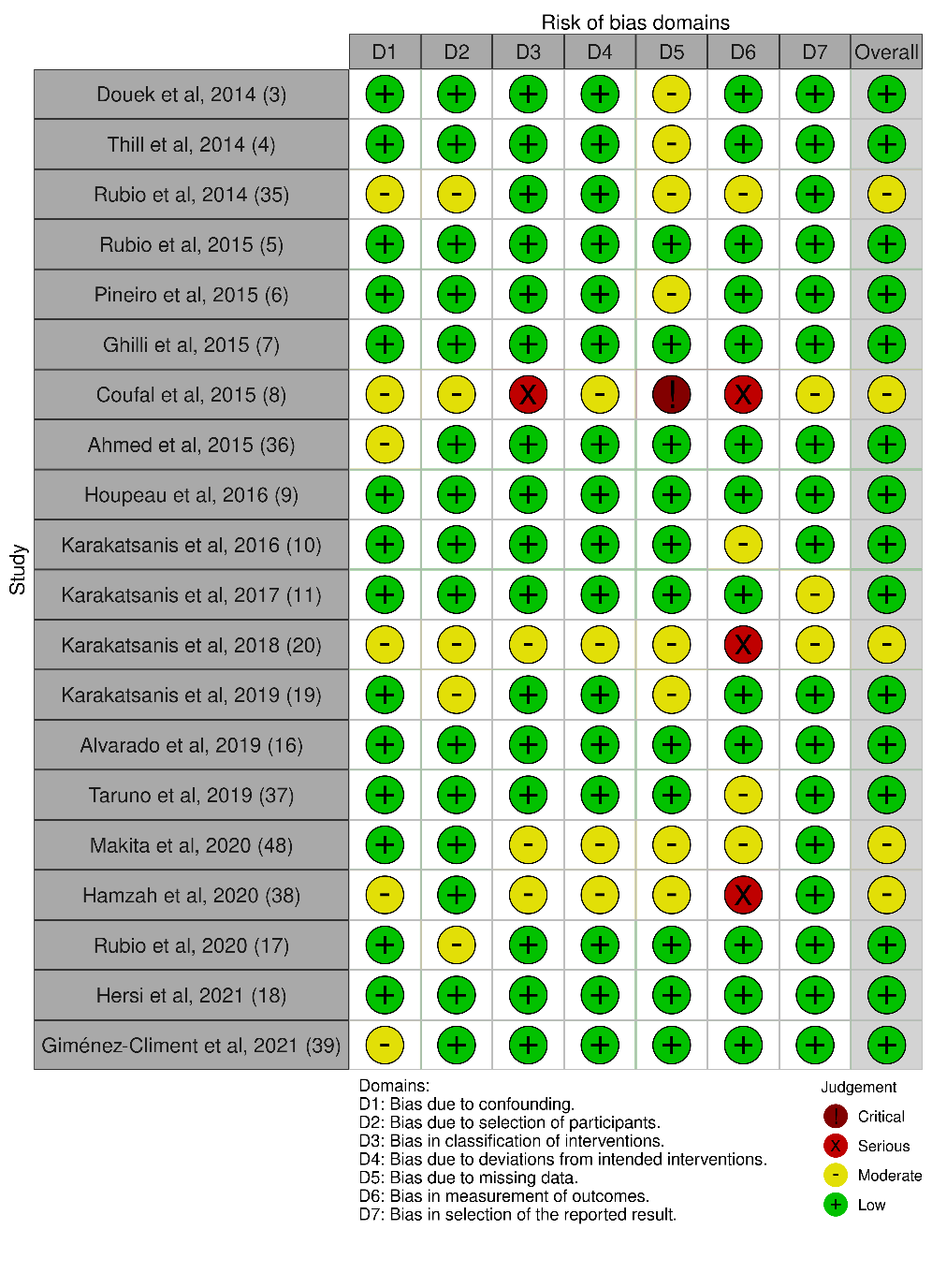
Risk-of-bias plot for the ROBINS-I tool of comparative studies

**1.b: Single-arm studies.**

1.b.(i): Detailed MINORS criteria

| **Study ID** | **Aim** | **Inclusion** | **Prospective** | **Endpoints** | **Endpoint assessment** | **Follow-up** | **Loss <5%** | **Sample size** | **Total** |
| --- | --- | --- | --- | --- | --- | --- | --- | --- | --- |
| **Hersi et al, 2019 (23)** | 2 | 2 | 2 | 2 | 2 | 2 | 2 | 0 | **14** |
| **Lorek et al, 2019 (42)** | 2 | 2 | 1 | 1 | 2 | 2 | 1 | 0 | **11** |
| **Man et al, 2019 (43)** | 2 | 2 | 1 | 2 | 2 | 2 | 2 | 0 | **13** |
| **Vural et al, 2019 (44)** | 2 | 2 | 2 | 2 | 2 | 2 | 2 | 0 | **14** |
| **Bazire et al 2019 (45)** | 2 | 1 | 0 | 2 | 2 | 1 | 1 | 0 | **9** |
| **Pohlodek et al, 2019 (46)** | 2 | 2 | 1 | 2 | 2 | 2 | 2 | 0 | **13** |
| **Kurylcio et al, 2021 (47)** | 2 | 2 | 1 | 1 | 1 | 1 | 2 | 0 | **10** |

1.b.(ii): Risk-of-Bias (RoB) assessment

| **Study ID** | **Sampling bias** | **Selection bias** | **Deviation from interventions bias** | **Missing data bias** | **Measurement of outcomes bias** | **Report bias** | **Overall** |
| --- | --- | --- | --- | --- | --- | --- | --- |
| **Hersi et al, 2019 (23)** | High | Low | Low | Some concerns | Some concerns | Some concerns | **Moderate** |
| **Lorek et al, 2019 (42)** | Some concerns | Low | Some concerns | High | High | No information | **High** |
| **Man et al, 2019 (43)** | Some concerns | Some concerns | Some concerns | Some concerns | High | Some concerns | **Moderate** |
| **Vural et al, 2019 (44)** | Some concerns | Some concerns | Low | No information | Some concerns | Low | **Moderate** |
| **Bazire et al 2019 (45)** | High | Low | No information | High | High | Low | **High** |
| **Pohlodek et al, 2019 (46)** | Some concerns | Some concerns | No information | Low | Some concerns | Some concerns | **Moderate** |
| **Kurylcio et al, 2021 (47)** | Some concerns | Some concerns | No information | No information | No information | High | **High** |


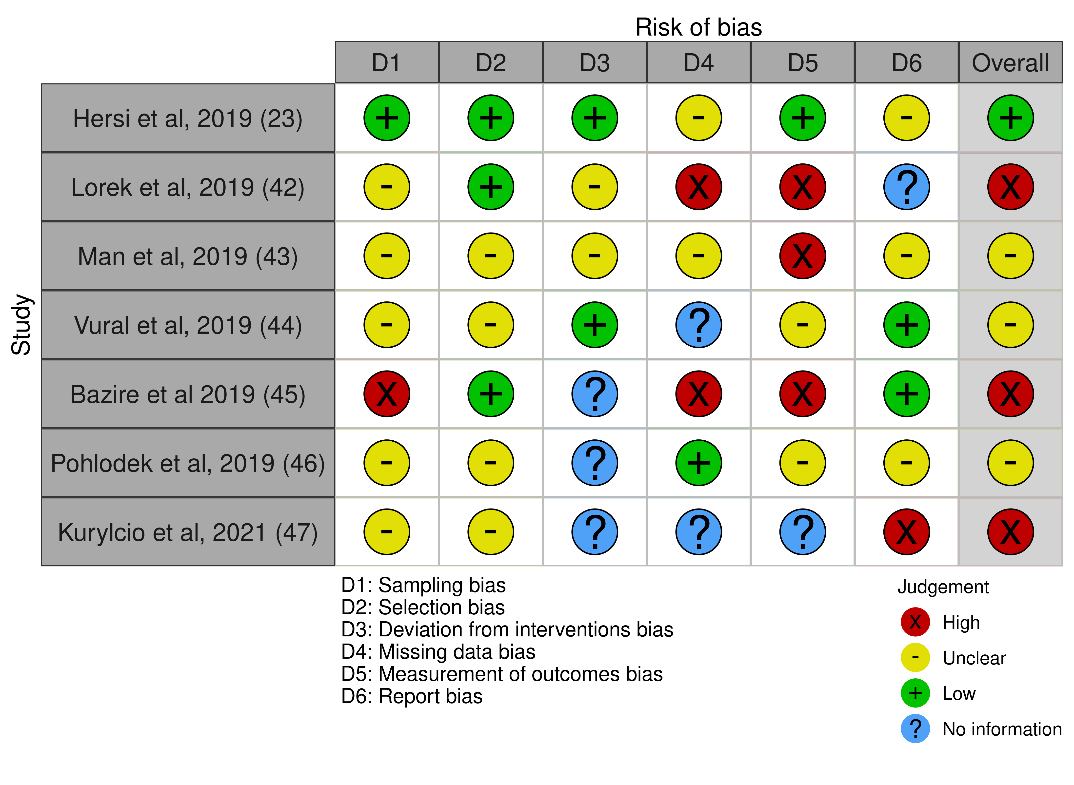
Risk-of-bias plot for single-arm studies

1. **Studies on MRI artifacts**

2.a: Newcastle-Ottawa Scale (NOS) criteria

| **Study ID** | **Selection** | | | | **Comparability** | **Outcome** | | | **Total score** |
| --- | --- | --- | --- | --- | --- | --- | --- | --- | --- |
|  | Representativeness of the exposed cohort | Selection of the non-exposed cohort | Ascertainment of exposure | Demonstration that outcome of interest was not present at start of study | Comparability | Standardised Assessment of outcome with independency | Adequacy of follow-up | Lost to follow-up (less than 10% and reported) |  |
| **Krischner et al, 2018 (12)** | -- | -- | * | * | -- | -- | * | -- | **3** |
| **Aribal et al, 2021 (13)** | -- | -- | * | * | -- | -- | * | -- | **3** |
| **Chapman et al, 2021 (14)** | -- | -- | * | * | -- | -- | * | -- | **3** |
| **Christenhuz et al, 2022 (15)** | -- | -- | * | * | * | * | * | -- | **5** |

2.b: QUADAS-2 tool

| **Study ID** | **Patient selection** | **Index test** | **Reference Standard** | **Flow and timing** | **Overall** |
| --- | --- | --- | --- | --- | --- |
| **Krischner et al, 2018 (12)** | High | High | No information | No information | High |
| **Aribal et al, 2021 (13)** | High | High | High | High | High |
| **Chapman et al, 2021 (14)** | High | High | No information | No information | High |
| **Christenhuz et al, 2022 (15)** | High | High | High | High | High |


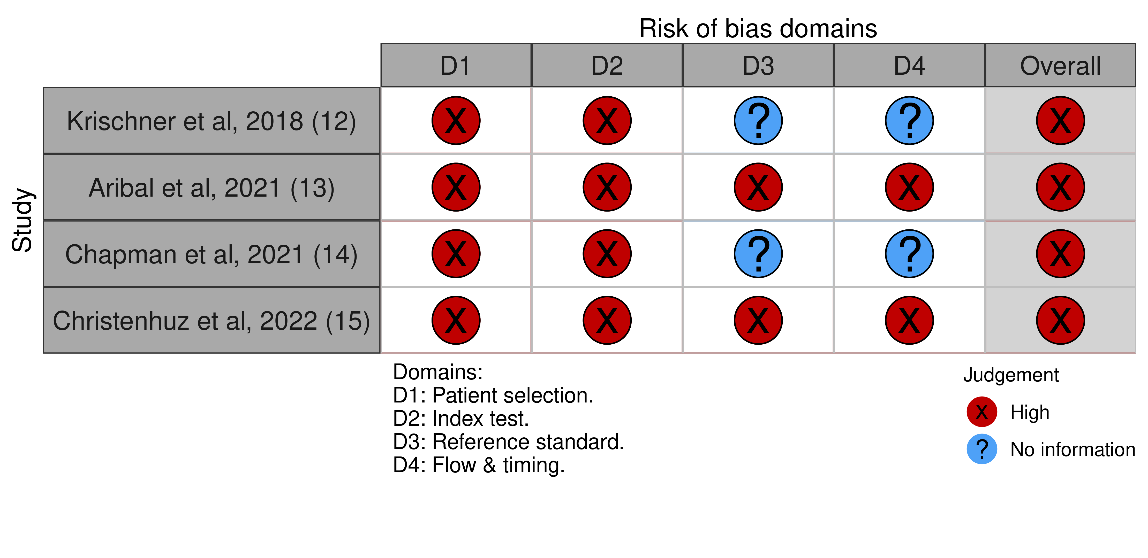
Risk-of-bias plot for the QUADAS-2 tool of studies for MRI artifacts

**Table S2: GRADE recommendations**

**Do Superparamagnetic Iron Oxide nanoparitcles (SPIO) perform comparably to Isotope with or without blue dye (RI+/-BD) for standard SLN detection in patients with breast cancer?**

| **Certainty assessment** | | | | | | | **№ of patients** | | **Effect** | | **Certainty** | **Importance** |
| --- | --- | --- | --- | --- | --- | --- | --- | --- | --- | --- | --- | --- |
| **№ of studies** | **Study design** | **Risk of bias** | **Inconsistency** | **Indirectness** | **Imprecision** | **Other considerations** | **Superparamagnetic Iron Oxide nanopartcles (SPIO)** | **Isotope with or without blue dye (RI+/-BD)** | **Relative (95% CI)** | **Absolute (95% CI)** |  |  |
| **Detection Rate** | | | | | | | | | | | | |
| 20 | observational studies | not serious | not serious | not serious | not serious | none | 2370/2430 (97.5%) | 2320/2404 (96.5%) | **RR 1.01** (0.99 to 1.02) | **10 more per 1.000** (from 10 fewer to 19 more) | ⨁⨁⨁⨁ High | IMPORTANT |
| **Concordance between SPIO and RI+/-BD** | | | | | | | | | | | | |
| 19 | observational studies | not serious | not serious | not serious | not serious | none | 2123/2144 (99.0%) | 2123/2186 (97.1%) | **Rate difference -0.003** (-0.009 to 0.015) | **-- per 1.000** (from -- to --) | ⨁⨁⨁⨁ High | IMPORTANT |
| **Number of SLNs** | | | | | | | | | | | | |
| 19 | observational studies | serious^a^ | serious^a^ | not serious | serious^a^ | strong association | 4201/4536 (92.6%)* | 3926/4592 (85.5%)* | **RR 1.10** (1.06 to 1.14) | **68 more per 1.000** (from 43 more to 94 more) | ⨁⨁◯◯ Low | NOT IMPORTANT |
|  |  |  |  |  |  |  | 94.1%  (pooled weighted rate) | 83.5%  (pooled weighted rate) |  | **67 more per 1.000** (from 42 more to 92 more) |  |  |

**CI:** confidence interval; **RR:** risk ratio, **SLN:** sentinel lymph node

#### Explanations

1. Heterogeneity in definition of SLN across studies (only tracer-active, tracer and/or BD active, tracer inactive but palpable, etc.). *: crude proportion rates

**Figure S1. PRISMA 2020 flow diagram for new systematic reviews which included searches of databases, registers and other sources**

**Identification of studies via other methods**

**Identification of studies via databases and registers**

Records identified from:

Websites (n =1 )

Organisations (n = 1)

Citation searching (n = 4)

Records removed *before screening*:

Duplicate records removed (n = 2)

Records identified from*:

Databases (n = 721)

**Identification**

Records screened

(n = 719)

Records excluded**

(n = 650)

Reports not retrieved

(n =0)

Reports sought for retrieval

(n = 5)

Reports sought for retrieval

(n = 69)

Reports not retrieved

(n =0 )

**Screening**

Reports excluded: 0

Reports assessed for eligibility

(n = 5)

Reports assessed for eligibility

(n = 69)

Reports excluded: 42

Imaging studies (n = 8)

Reviews (n = 12)

Animal studies (n = 5)

Letters to editor (n= 7)

Other cancers (n= 10)

Studies included in review

(n = 32)

Reports of included studies

(n = 32)

**Included**

**Figure S2: Forest plot for Detection rate (per patient/procedure)**

**
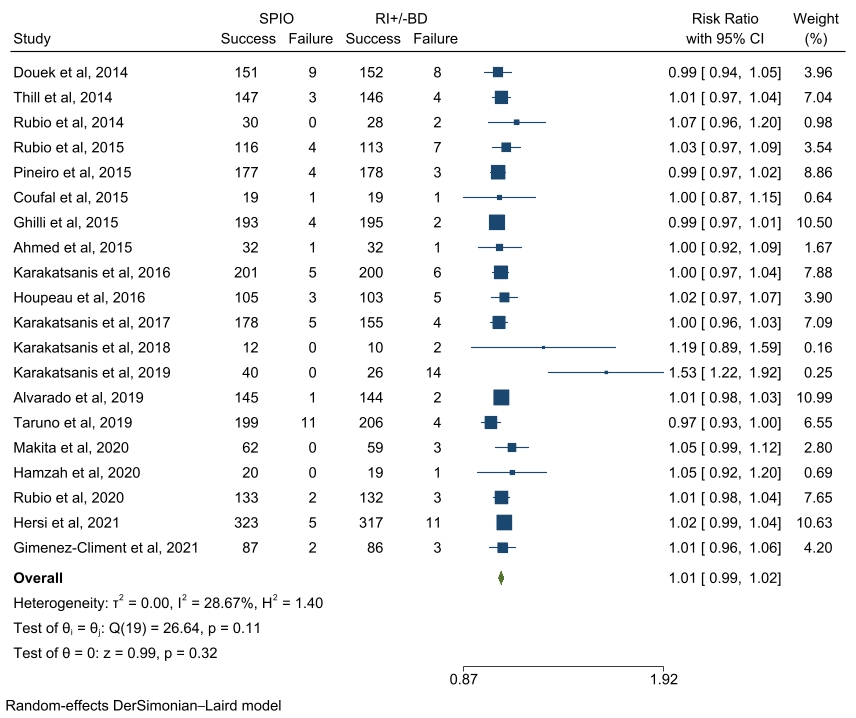
**

**Figure S3: Forest plot for Nodal detection rate**

**
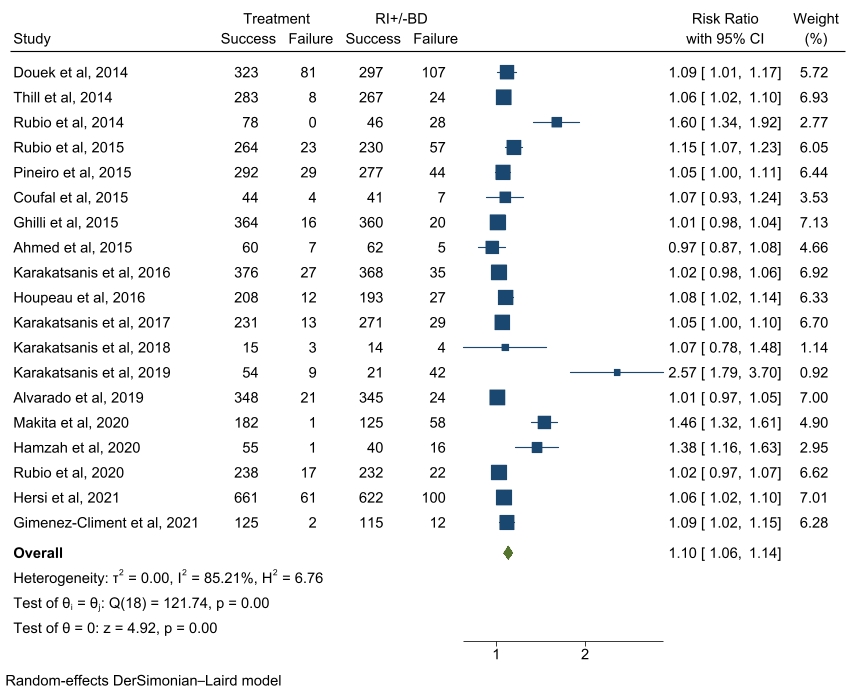
**

**Figure S4: Forest plot for Difference between Concordance and Reverse Concordance**

**
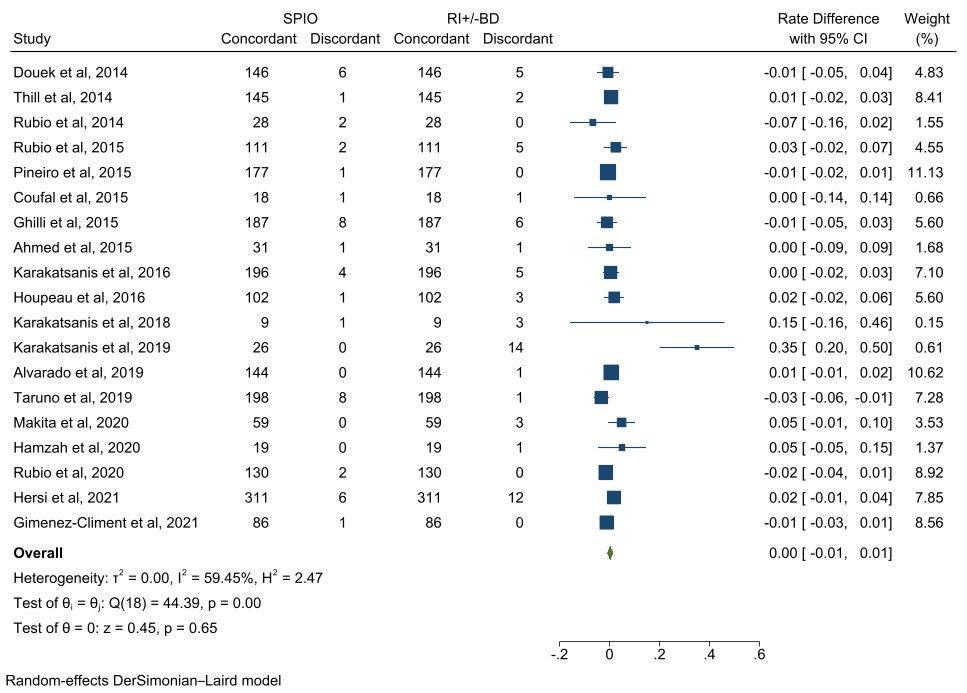
**

**Figure S5: Forest plot for SPIO-induced skin staining**

**
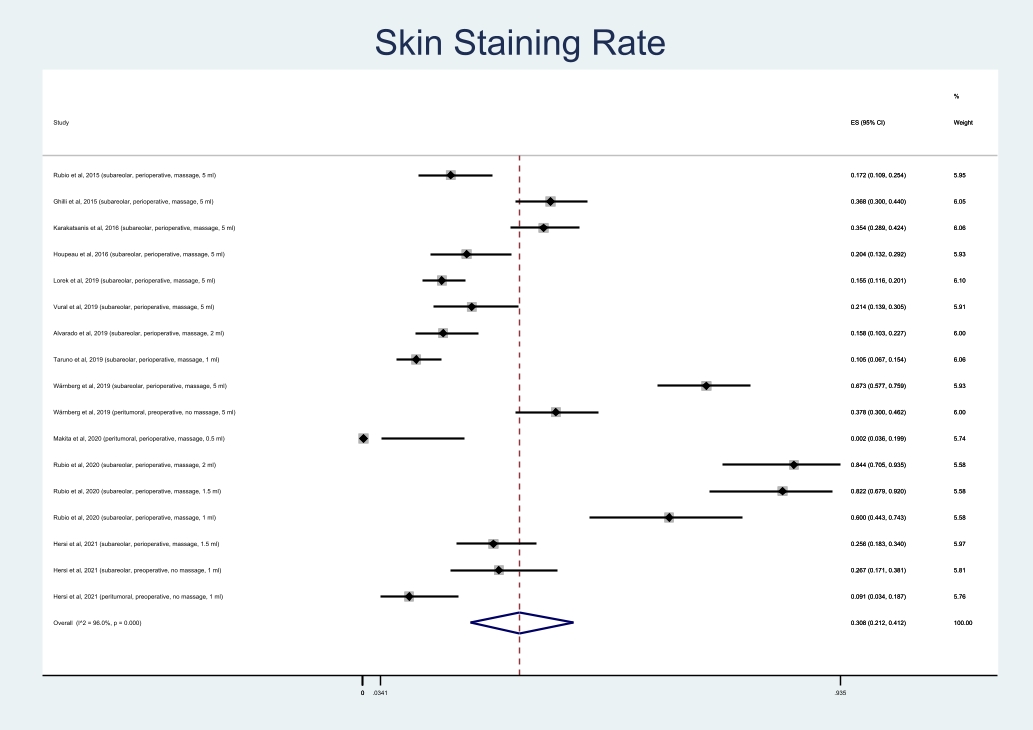
**

# PRISMA 2020 Main Checklist

| **Topic** | **No.** | **Item** | **Location where item is reported** |
| --- | --- | --- | --- |
| **TITLE** |  |  |  |
| **Title** | 1 | Identify the report as a systematic review. | Page 1, Row 1-2 |
| **ABSTRACT** |  |  |  |
| **Abstract** | 2 | See the PRISMA 2020 for Abstracts checklist |  |
| **INTRODUCTION** |  |  |  |
| **Rationale** | 3 | Describe the rationale for the review in the context of existing knowledge. | Page 3, Row 86-98 |
| **Objectives** | 4 | Provide an explicit statement of the objective(s) or question(s) the review addresses. | Page 3, Rows 99-102 |
| **METHODS** |  |  |  |
| **Eligibility criteria** | 5 | Specify the inclusion and exclusion criteria for the review and how studies were grouped for the syntheses. | Page 4, Rows 136-146 |
| **Information sources** | 6 | Specify all databases, registers, websites, organisations, reference lists and other sources searched or consulted to identify studies. Specify the date when each source was last searched or consulted. | Page 4, Rows 136-146 |
| **Search strategy** | 7 | Present the full search strategies for all databases, registers and websites, including any filters and limits used. | Page 4, Rows 136-146 |
| **Selection process** | 8 | Specify the methods used to decide whether a study met the inclusion criteria of the review, including how many reviewers screened each record and each report retrieved, whether they worked independently, and if applicable, details of automation tools used in the process. | Page 4, Rows 136-146 |
| **Data collection process** | 9 | Specify the methods used to collect data from reports, including how many reviewers collected data from each report, whether they worked independently, any processes for obtaining or confirming data from study investigators, and if applicable, details of automation tools used in the process. | Page 4, Rows 149-150 |
| **Data items** | 10a | List and define all outcomes for which data were sought. Specify whether all results that were compatible with each outcome domain in each study were sought (e.g. for all measures, time points, analyses), and if not, the methods used to decide which results to collect. | Pages 4-5, Rows 151-164 |
|  | 10b | List and define all other variables for which data were sought (e.g. participant and intervention characteristics, funding sources). Describe any assumptions made about any missing or unclear information. | Pages 4-5, Rows 151-164 |
| **Study risk of bias assessment** | 11 | Specify the methods used to assess risk of bias in the included studies, including details of the tool(s) used, how many reviewers assessed each study and whether they worked independently, and if applicable, details of automation tools used in the process. | Supplement Table 1 |
| **Effect measures** | 12 | Specify for each outcome the effect measure(s) (e.g. risk ratio, mean difference) used in the synthesis or presentation of results. | Pages 4-5, Rows 151-164 |
| **Synthesis methods** | 13a | Describe the processes used to decide which studies were eligible for each synthesis (e.g. tabulating the study intervention characteristics and comparing against the planned groups for each synthesis (item 5)). | Pages 4-5, Rows 151-164 |
|  | 13b | Describe any methods required to prepare the data for presentation or synthesis, such as handling of missing summary statistics, or data conversions. | Pages 4-5, Rows 151-164 |
|  | 13c | Describe any methods used to tabulate or visually display results of individual studies and syntheses. | Pages 4-5, Rows 151-164 |
|  | 13d | Describe any methods used to synthesize results and provide a rationale for the choice(s). If meta-analysis was performed, describe the model(s), method(s) to identify the presence and extent of statistical heterogeneity, and software package(s) used. | Pages 4-5, Rows 151-164 |
|  | 13e | Describe any methods used to explore possible causes of heterogeneity among study results (e.g. subgroup analysis, meta-regression). | Pages 4-5, Rows 151-164 |
|  | 13f | Describe any sensitivity analyses conducted to assess robustness of the synthesized results. | Pages 4-5, Rows 151-164 |
| **Reporting bias assessment** | 14 | Describe any methods used to assess risk of bias due to missing results in a synthesis (arising from reporting biases). | Page 5, Rows 166-176 |
| **Certainty assessment** | 15 | Describe any methods used to assess certainty (or confidence) in the body of evidence for an outcome. | Page 4, Rows 131-133 |
| **RESULTS** |  |  |  |
| **Study selection** | 16a | Describe the results of the search and selection process, from the number of records identified in the search to the number of studies included in the review, ideally using a flow diagram. | Page 5, Rows 179-198; Figure 1 (PRISMA flowchart) |
|  | 16b | Cite studies that might appear to meet the inclusion criteria, but which were excluded, and explain why they were excluded. | No such studies were found |
| **Study characteristics** | 17 | Cite each included study and present its characteristics. | Table 1; Table 2 |
| **Risk of bias in studies** | 18 | Present assessments of risk of bias for each included study. | Supplement Table 1 |
| **Results of individual studies** | 19 | For all outcomes, present, for each study: (a) summary statistics for each group (where appropriate) and (b) an effect estimate and its precision (e.g. confidence/credible interval), ideally using structured tables or plots. | Table 1; Table 2 |
| **Results of syntheses** | 20a | For each synthesis, briefly summarise the characteristics and risk of bias among contributing studies. | Page 5-8, Rows 200-293 |
|  | 20b | Present results of all statistical syntheses conducted. If meta-analysis was done, present for each the summary estimate and its precision (e.g. confidence/credible interval) and measures of statistical heterogeneity. If comparing groups, describe the direction of the effect. | Page 5-8, Rows 200-293 |
|  | 20c | Present results of all investigations of possible causes of heterogeneity among study results. | Page 5-8, Rows 200-293 |
|  | 20d | Present results of all sensitivity analyses conducted to assess the robustness of the synthesized results. | Page 5-8, Rows 200-293 |
| **Reporting biases** | 21 | Present assessments of risk of bias due to missing results (arising from reporting biases) for each synthesis assessed. | Supplement Table 1 |
| **Certainty of evidence** | 22 | Present assessments of certainty (or confidence) in the body of evidence for each outcome assessed. | Page 8-9, Rows 295-336 |
| **DISCUSSION** |  |  |  |
| **Discussion** | 23a | Provide a general interpretation of the results in the context of other evidence. | Page 1, Rows 340-354 |
|  | 23b | Discuss any limitations of the evidence included in the review. | Page 9, Rows 369-370; Page 9-10, Rows 375-376 |
|  | 23c | Discuss any limitations of the review processes used. | Page 10, Rows 389-394 |
|  | 23d | Discuss implications of the results for practice, policy, and future research. | Page 10, Rows 394-401 |
| **OTHER INFORMATION** |  |  |  |
| **Registration and protocol** | 24a | Provide registration information for the review, including register name and registration number, or state that the review was not registered. | Page 10; Rows 404-412 |
|  | 24b | Indicate where the review protocol can be accessed, or state that a protocol was not prepared. | Page 10; Rows 404-412 |
|  | 24c | Describe and explain any amendments to information provided at registration or in the protocol. | Not relevant as not performed |
| **Support** | 25 | Describe sources of financial or non-financial support for the review, and the role of the funders or sponsors in the review. | Page 10; Rows 404-412 |
| **Competing interests** | 26 | Declare any competing interests of review authors. | Page 10; Rows 404-412 |
| **Availability of data, code and other materials** | 27 | Report which of the following are publicly available and where they can be found: template data collection forms; data extracted from included studies; data used for all analyses; analytic code; any other materials used in the review. | Page 10; Rows 404-412 |

#####

# PRIMSA Abstract Checklist

| **Topic** | **No.** | **Item** | **Reported?** |
| --- | --- | --- | --- |
| **TITLE** |  |  |  |
| **Title** | 1 | Identify the report as a systematic review. | Yes |
| **BACKGROUND** |  |  |  |
| **Objectives** | 2 | Provide an explicit statement of the main objective(s) or question(s) the review addresses. | Yes |
| **METHODS** |  |  |  |
| **Eligibility criteria** | 3 | Specify the inclusion and exclusion criteria for the review. | No |
| **Information sources** | 4 | Specify the information sources (e.g. databases, registers) used to identify studies and the date when each was last searched. | No |
| **Risk of bias** | 5 | Specify the methods used to assess risk of bias in the included studies. | Yes |
| **Synthesis of results** | 6 | Specify the methods used to present and synthesize results. | Yes |
| **RESULTS** |  |  |  |
| **Included studies** | 7 | Give the total number of included studies and participants and summarise relevant characteristics of studies. | Yes |
| **Synthesis of results** | 8 | Present results for main outcomes, preferably indicating the number of included studies and participants for each. If meta-analysis was done, report the summary estimate and confidence/credible interval. If comparing groups, indicate the direction of the effect (i.e. which group is favoured). | Yes |
| **DISCUSSION** |  |  |  |
| **Limitations of evidence** | 9 | Provide a brief summary of the limitations of the evidence included in the review (e.g. study risk of bias, inconsistency and imprecision). | Yes |
| **Interpretation** | 10 | Provide a general interpretation of the results and important implications. | Yes |
| **OTHER** |  |  |  |
| **Funding** | 11 | Specify the primary source of funding for the review. | Yes |
| **Registration** | 12 | Provide the register name and registration number. | No |

*From:* Page MJ, McKenzie JE, Bossuyt PM, Boutron I, Hoffmann TC, Mulrow CD, et al. The PRISMA 2020 statement: an updated guideline for reporting systematic reviews. MetaArXiv. 2020, September 14. DOI: 10.31222/osf.io/v7gm2. For more information, visit: [www.prisma-statement.org](file:///C:\Users\andka774\Downloads\www.prisma-statement.org)
